# Validated limited gene predictor for cervical cancer lymph node metastases

**DOI:** 10.18632/oncotarget.27632

**Published:** 2020-06-16

**Authors:** Joshua D. Bloomstein, Rie von Eyben, Andy Chan, Erinn B. Rankin, Daniel R. Fregoso, Jing Wang-Chiang, Lisa Lee, Liang-Xi Xie, Shannon MacLaughlan David, Henning Stehr, Mohammad S. Esfahani, Amato J. Giaccia, Elizabeth A. Kidd

**Affiliations:** ^1^Department of Radiation Oncology, Stanford University School of Medicine, Stanford, CA, USA; ^2^Department of Gynecologic Oncology, Santa Clara Valley Medical Center, Fruitdale, CA, USA; ^3^Department of Radiation Oncology, Xiamen University Xiang’an Hospital, Xiamen, Fujian, China; ^4^Department of Clinical Obstetrics & Gynecology, University of Illinois, Chicago, IL, USA; ^5^Department of Pathology, Stanford University School of Medicine, Stanford, CA, USA

**Keywords:** cervical cancer, metastasis

## Abstract

Purpose: Recognizing the prognostic significance of lymph node (LN) involvement for cervical cancer, we aimed to identify genes that are differentially expressed in LN+ versus LN- cervical cancer and to potentially create a validated predictive gene signature for LN involvement.

Materials and Methods: Primary tumor biopsies were collected from 74 cervical cancer patients. RNA was extracted and RNA sequencing was performed. The samples were divided by institution into a training set (*n* = 57) and a testing set (*n* = 17). Differentially expressed genes were identified among the training cohort and used to train a Random Forest classifier.

Results: 22 genes showed > 1.5 fold difference in expression between the LN+ and LN- groups. Using forward selection 5 genes were identified and, based on the clinical knowledge of these genes and testing of the different combinations, a 2-gene Random Forest model of BIRC3 and CD300LG was developed. The classification accuracy of lymph node (LN) status on the test set was 88.2%, with an Area under the Receiver Operating Characteristic curve (ROC-AUC) of 98.6%.

Conclusions: We identified a 2 gene Random Forest model of BIRC3 and CD300LG that predicted lymph node involvement in a validation cohort. This validated model, following testing in additional cohorts, could be used to create a reverse transcription-quantitative polymerase chain reaction (RT-qPCR) tool that would be useful for helping to identify patients with LN involvement in resource-limited settings.

## INTRODUCTION

Cervical cancer is the 4th most common cause of cancer death in women worldwide [1]. Metastasis is a central cause of mortality in cervical cancer, and patients with lymph node involvement are more likely to progress to have distant metastases. Patients with lymph node involvement have significantly worse 5-year overall survival compared to those with localized disease only [2]. Currently lymph node involvement is usually determined by surgical pathology or imaging, such as FDG-PET/CT or MRI. To date there is no simple lab test that accurately predicts which patients will progress to have lymph node involvement. Unfortunately, the incidence of cervical cancer is increasing in developing countries with limited health care resources. A pathologic tool that could help stratify patients based on lymph node status could be particularly beneficial for determining the best utilization of treatment resources. Primary cervical tumor biopsy is relatively non-invasive, and nucleic acid isolates can be sequenced fairly quickly and inexpensively. An RNA-seq-based signature, therefore, is a sensible candidate method for development of a model that predicts lymph node involvement.

## RESULTS

Patient and cervical cancer characteristics are included in Table 1 with 54% of patients with lymph node metastases. Based on primary tumor pathology, 86% of patients had squamous cell carcinoma, and the remainder had either adenocarcinoma or adenosquamous.

**Table 1:** Characteristics of patients with cervical cancer in the three cohorts–breakdown of patient groups by LN status, histology, and tumor stage

Characteristic		Stanford Medical Center (*N* = 47)	Cancer Hospital of Shantou Medical Center (*N* = 10)	Training Cohort (*N* = 57)	Validation Cohort: Santa Clara Valley Medical Center (*N* = 17)
Lymph node status - no. (%)	Positive	30 (64)	2 (20)	32 (56)	8 (47)
	Negative	17 (36)	8 (80)	25 (44)	9 (53)
Positive lymph node location - no.	Pelvic	22	1	23	4
	Para-aortic	8	1	9	4
Lymph node status method - no.	PET/CT	38	1	39	11
	Histology	9	9	18	6
Histology - no. (%)	Squamous cell carcinoma	43 (92)	10 (100)	53 (93)	11 (65)
	Adenocarcinoma	3 (6)	0 (0)	3 (5)	4 (24)
	Adenosquamous	1 (2)	0 (0)	1 (2)	2 (12)
FIGO 2008 Stage - no. (%)	I	10 (21)	6 (60)	16 (28)	4 (24)
	II	25 (53)	4 (40)	29 (51)	6 (35)
	III	8 (17)	0 (0)	8 (14)	4 (24)
	IV	4 (9)	0 (0)	4 (7)	3 (18)

LN status determined by pathology or PET/CT imaging. Tumor stage corresponds to FIGO 2009 staging.

**Figure 1:**
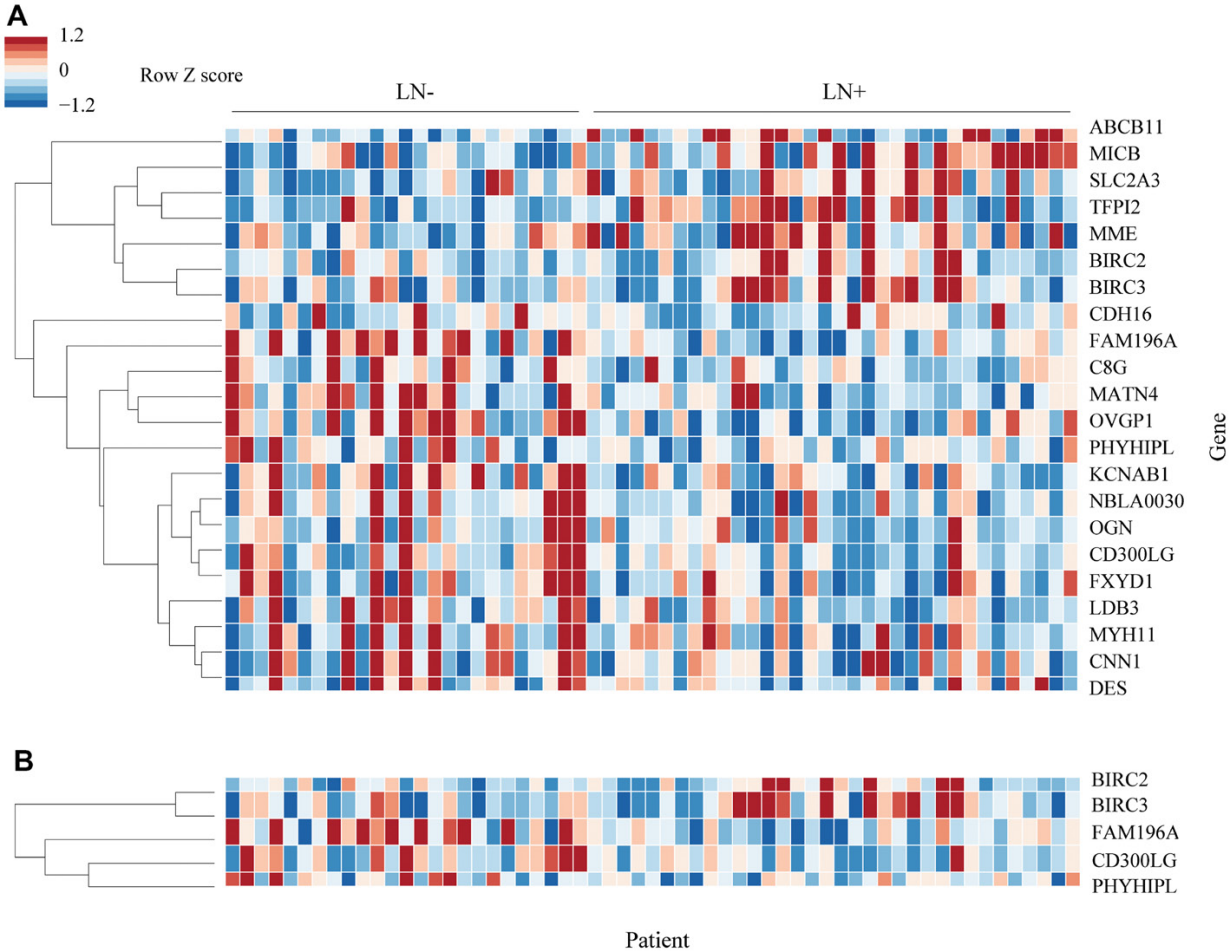
Heatmap of differentially expressed genes. (**A**) 22 genes were differentially expressed in the training set. Genes were arranged by hierarchical clustering using correlation distance and average linkage. Fold-change is expressed in linear scale as Z score across samples. (**B**) 5 genes were selected from the original 22 by forward selection. Clustering for 5 gene heatmap was performed separately.

Using the training set of 57 samples, genes that were differentially expressed between LN+ patients and LN- were identified. 22 genes showed 1.5 or greater fold difference in expression between the groups with FDR ≤ 0.01 (Table 2). A heatmap of the differentially expressed genes is shown in Figure 1.

**Table 2:** Differentially expressed genes in the training set–22 genes in the training set were differentially expressed, which was defined as fold-change > 1.5 and FDR ≤ 0.01

Gene Symbol	Gene Name	Fold-Change (LN+/LN–)	FDR	Gene Symbol
OGN	Osteoglycin	0.605	0.001	OGN
DES	Desmin	0.564	0.001	DES
CD300LG	CD300 Molecule Like Family Member G	0.658	0.001	CD300LG
C8G	Complement C8 Gamma Chain	0.553	0.002	C8G
OVGP1	Oviductal Glycoprotein 1	0.580	0.003	OVGP1
SLC2A3	Solute Carrier Family 2 Member 3	1.757	0.003	SLC2A3
CDH16	Cadherin 16	0.598	0.004	CDH16
FAM196A	Inhibitory Synaptic Factor 2A	0.568	0.004	FAM196A
LDB3	LIM Domain Binding 3	0.568	0.004	LDB3
MME	Membrane Metalloendopeptidase	1.656	0.004	MME
MYH11	Myosin Heavy Chain 11	0.575	0.004	MYH11
NBLA00301	HAND2 Antisense RNA 1	0.601	0.004	NBLA00301
TFPI2	Tissue Factor Pathway Inhibitor 2	2.050	0.004	TFPI2
KCNAB1	Potassium Voltage-Gated Channel Subfamily A Member Regulatory Beta Subunit 1	0.591	0.006	KCNAB1
MATN4	Matrilin 4	0.580	0.007	MATN4
ABCB11	ATP Binding Cassette Subfamily B Member 11	1.605	0.008	ABCB11
BIRC3	Baculoviral Iap Repeat Containing 3	1.664	0.008	BIRC3
FXYD1	FXYD Domain Containing Ion Transport Regulator 1	0.588	0.008	FXYD1
BIRC2	Baculoviral Iap Repeat Containing 2	1.677	0.009	BIRC2
MICB	MHC Class I Polypeptide-Related Sequence B	1.653	0.009	MICB
PHYHIPL	Phytanoyl-CoA 2-Hydroxylase Interacting Protein Like	0.598	0.010	PHYHIPL
CNN1	Calponin 1	0.597	0.010	CNN1

Fold-change is represented on linear scale. Genes are ranked by increasing FDR.

**Figure 2:**
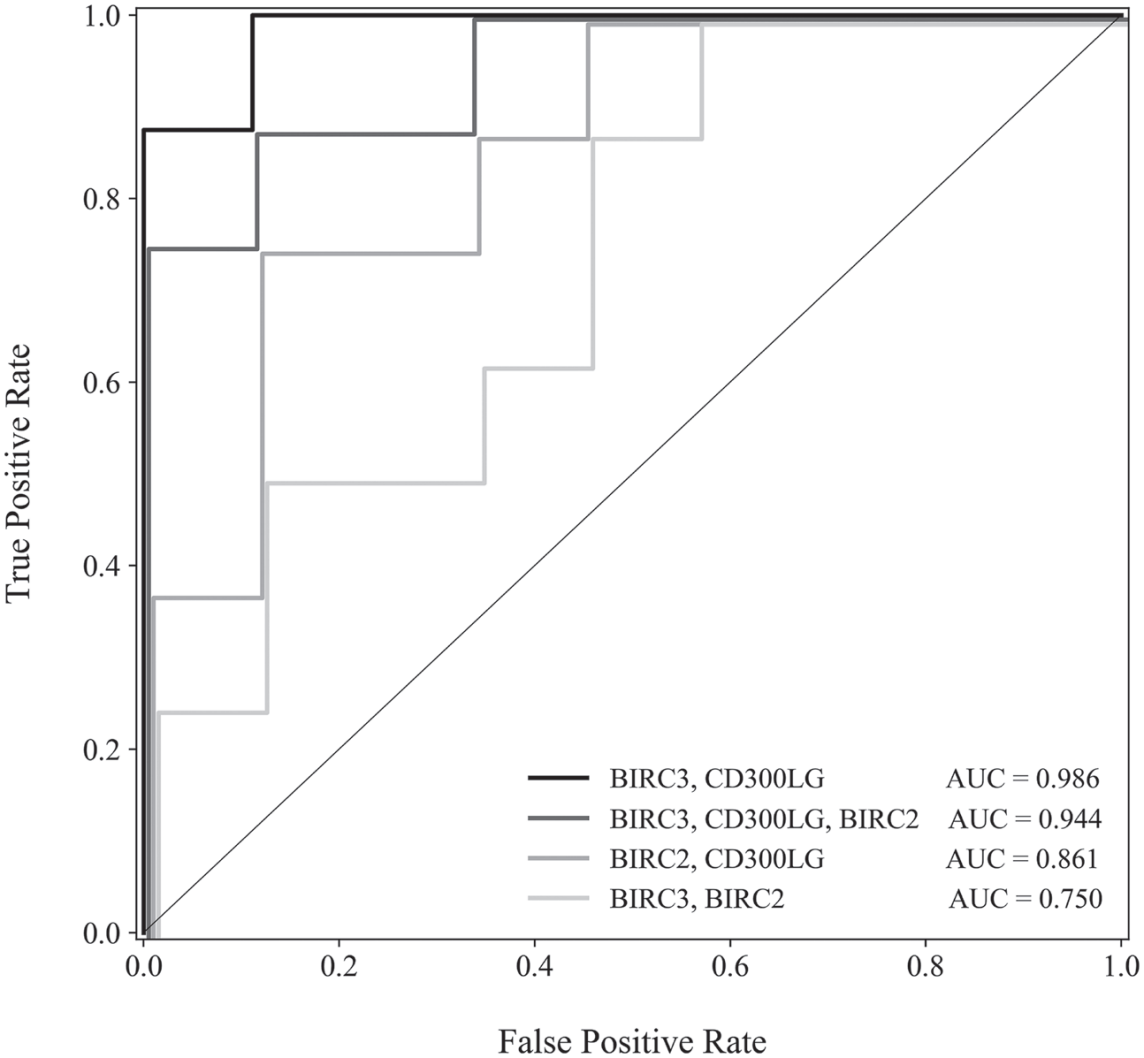
Receiver operating characteristic curve comparing 4 models–models based on each combination of the top 3 genes were evaluated for sensitivity and specificity. Receiver operating characteristic curve displays false positive rate (1 – specificity) versus true positive rate (sensitivity).

**Figure 3:**
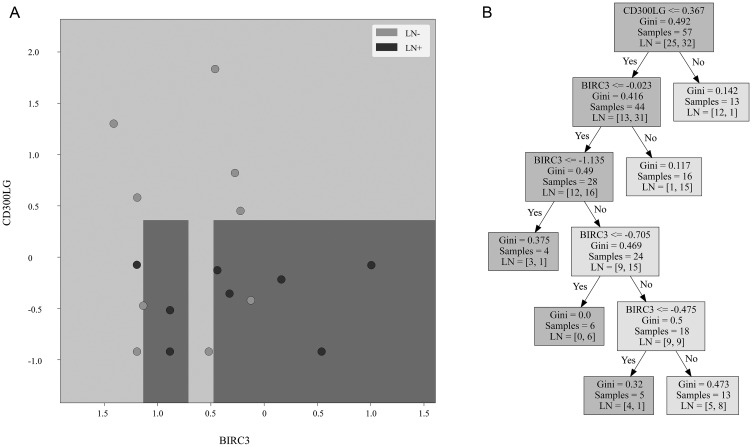
Decision surface and decision tree. (**A**) Decision surface of a decision tree derived from the training cohort using the same genes as the Random Forest model. The background is the decision boundary generated by the decision tree based on the training cohort data. Light color represents the prediction of lymph node negative sample, and dark color represents prediction of lymph node positive sample. The validation cohort samples are overlaid. Light samples are lymph node negative tumors and dark samples are lymph node positive tumors. (**B**) Decision tree created from the training cohort using the same genes.

Out of the 22 differentially expressed genes, 5 were identified as potential candidates for the predictive model using forward selection: BIRC2, BIRC3, CD300LG, FAM196A, and PHYHIPL. 2 of these genes, FAM196A and PHYHIPL, have very limited gene annotation or relevant clinical background and were therefore excluded from model development. The final three genes, BIRC2, BIRC3, and CD300LG, were included in the training set Random Forest model. The subset of BIRC3 and CD300LG showed the best test set accuracy, 88.2%, and was therefore selected as the final model. In the training set, BIRC3 (Baculoviral IAP Repeat Containing 3) was upregulated 1.7-fold in LN+ patients. CD300LG (Nepmucin), on the other hand, was downregulated 1.5-fold in LN+ patients. Table 3 compares the accuracy of the various models tested, while Figure 2 shows the ROC curve comparison of the 4 models. Figure 3 shows the Random Forest decision surface and a decision tree using the 2 genes with the training set.

**Table 3:** Comparison of lymph node prediction accuracies of potential models – each combination of the top 3 genes was used to develop a Random Forest model

Model	Accuracy training cohort (*N* = 57)	ROC-AUC [95% CI] training cohort	Accuracy test cohort (*N* = 17)	ROC-AUC [95% CI] test cohort
BIRC3, CD300LG	87.7	97.0 [90.4, 99.6]	88.2	98.6 [86.1, 100]
BIRC3, CD300LG, BIRC2	87.7	98.5 [94.2, 100]	82.4	94.4 [71.6, 100]
BIRC2, CD300LG	87.7	98.2 [92.5, 100]	70.6	86.1 [51.7, 100]
BIRC3, BIRC2	93.0	98.9 [94.6, 100]	64.7	75.0 [40.3, 95.7]

Models are ranked by test set classification accuracy.

Alternative models were developed using the final 2 genes with different algorithms, but these models showed slightly lower performance than the Random Forest model. A Support Vector Machine with Radial Basis Function (RBF) kernel had 94.4% ROC-AUC, and a Gaussian Naïve Bayes model had 84.7% ROC-AUC.

The Random Forest model correctly classified 88.2% of the test set patients, *n* = 17. All 8 of the LN+ patients were correctly classified; out of the 9 LN- patients, 7 were correctly classified. The test set ROC-AUC was 98.6%, 95% CI [66.7%, 100%]. Sensitivity for detection of LN involvement was 100%, and specificity was 77.8%. Precision was 80.0% and Negative Predictive Potential was 100%.

## DISCUSSION

Lymph node involvement represents one of the most significant determinants of cervical cancer prognosis and impacts treatment approach [3–6]. Our pilot study shows that lymph node involvement in cervical cancer can be predicted with RNA-seq data, and our two-gene lymph node predictive signature showed 88% predictive accuracy when evaluated in a separate cohort.

Cervical cancer lymph node involvement can be determined by surgical staging or imaging, such as FDG-PET/CT or MRI. Several studies suggest that lymph node status on FDG-PET is a more significant predictor of disease outcome than clinical FIGO stage [6–8]. The presence of lymph nodes also influences treatment decisions, such as the need for adjuvant chemoradiation after surgery or the extent of the radiation field and dose for definitive chemoradiation treatment. Currently, no simple lab test that accurately predicts cervical cancer lymph node involvement exists. Additionally, the incidence of cervical cancer is increasing in developing countries with limited health care resources for surgery, imaging, and treatment. A simple pathologic tool that could help stratify patients based on predicted lymph node status could be particularly beneficial for determining the best utilization of treatment resources. If treatment or radiation resources are particularly limited, knowing which patients have no lymph nodes could help identify the patients to treat with a definitive or curative approach versus those to treat more palliatively. Alternatively, if imaging resources are limited but surgical and/or radiation resources are available, it might be that patients with a biomarker predicting for lymph nodes undergo surgical lymph node resection and/or extended field radiation or additional chemotherapy.

A limited number of microarray gene expression studies have been performed involving cervical cancer, and a few groups have attempted to identify a gene expression signature that can predict lymph node involvement in cervical cancer [9–12]. Many of these studies were relatively small and lacked a validation cohort. Grigsby et al., for example, included 8 cervical cancer patients, 3 with supraclavicular metastases, and identified 75 out of 12,000 genes with at least 3-fold differential expression to create a 12 gene signature to distinguish the groups [9]. Biewenga et al. evaluated tumor samples from 35 patients (16 with lymph node metastases) and found that 5 genes with differential expression had a prediction accuracy of 64.5% [10]. Using 43 primary cervical cancer samples (16 with lymph node metastases), Kim et al. created a lymph node prediction model using 156 genes with a prediction accuracy of 77% [11]. While Huang and colleagues did include a validation cohort, they evaluated early stage cervical cancer patients undergoing hysterectomy with fairly low rates of lymph node metastases [12]. In contrast, our study includes a range of stages, a greater proportion of advanced stage, a higher proportion with lymph node metastases, and also used deep sequencing with RNA-seq that assayed over 16,000 genes, as opposed to microarrays with fewer than 1,500 genes. Unfortunately, these different microarray studies for cervical cancer had minimal overlap in significant genes, suggesting that a new, more reproducible approach with RNA-seq might be a better method for a lymph node predictive model.

In benchmarking studies, RNA-seq has been shown to outperform microarray for both sensitivity and specificity [13–17], and therefore gene signatures derived from RNA-seq studies are likely to have greater extrinsic validity compared with those derived from microarray studies. RNA-seq is a direct high-throughput sequencing method that does not rely on hybridization, in contrast to microarray.

Among the 20 proteins corresponding to the differentially expressed genes with UniProt Gene Ontology annotation, 18 have annotation of integral membrane component, extracellular, ion/solute transport, or surface receptor (http://www.geneontology.org/). Considering that membrane proteins have key roles in the cellular changes that contribute to adhesion, epithelial-mesenchymal transition (EMT), and metastasis, these genes could potentially be linked to lymph node involvement.

BIRC3, one of the significant genes of our model, has an important role in the inhibition of apoptosis and could be related to the survival of cervical cancer cells during metastasis. In The Cancer Genome Atlas (TCGA) comprehensive characterization of cervical cancer, BIRC3 was highlighted for showing amplification events in 17% of tumors [18]. These amplification events were enriched in a cluster of primarily squamous tumors, which is similar to our dataset, composed of nearly all squamous tumors. While BIRC3 and CD300LG may play a role in the pathophysiology of cervical cancer metastasis, our gene expression study simply suggests a correlative connection. Further study of these genes in the context of cervical cancer progression is warranted.

A gene signature composed of two genes offers important advantages compared to larger gene signatures for the purpose of prediction. A smaller model is less likely to overfit to the training cohort, and its operation is less computationally expensive. A smaller gene set, moreover, may focus on fewer pathways or networks, potentially contributing to a more coherent interpretation of the biological factors involved. Additionally, a small panel is easier to assay in follow-up studies and could possibly translate into a more tractable clinical test, such as a reverse transcription-quantitative polymerase chain reaction (RT-qPCR) assay.

While our study has several unique and valuable findings, it also has some limitations. Our study included a range of stages and histologies, so stage IV and adenocarcinoma are included in more limited numbers. Patients were only assessed for lymph node status and were not uniformly followed for disease outcomes. The significant genes of our study have limited overlap with existing cervical cancer microarray studies.

Our study shows that lymph node involvement in cervical cancer can be predicted with RNA-seq data, and our limited gene lymph node predictive signature shows high predictive accuracy when evaluated in a separate cohort. Our study has several strengths, including the relatively large number of patients, the use of RNA-seq for the evaluation of gene expression, the validation of the predictive model with a separate cohort, and the high classification accuracy of lymph node status in the testing cohort. Further evaluating our findings in additional cohorts would be beneficial. In particular, a follow-up study involving survival data could assess the ability to predict clinical outcomes. Upon further validation, this biomarker tool that could predict lymph node involvement based on cervical tumor biopsy could be useful for risk stratification of cervical cancer patients in developing countries with limited imaging and healthcare resources.

## MATERIALS AND METHODS

### Tissue collection

With IRB approval 74 cervical biopsy samples were prospectively collected from cervical cancer patients, prior to the initiation of treatment or at the time of initial surgery. Inclusion criteria was for patients with known or suspected cervical cancer. If diagnostic and research biopsy were obtained at the same time and diagnostic biopsy found that the patient did not have cervical cancer, the patient and tissue sample were excluded from analysis. Additional clinical information prospectively collected included tumor stage, histology, and location of local lymph node involvement. The patient cancers mainly consist of squamous cell carcinomas (Table 1). Lymph node status was assessed by a combination of pathology (*n* = 24) and diagnostic FDG-PET/CT imaging (*n* = 50). Patients who underwent surgical assessment of lymph nodes generally had a pelvic lymph node dissection with or without para-aortic lymph node sampling. Lymph nodes were considered positive on FDG-PET/CT based on the interpretation by the nuclear medicine physicians. Clinical characteristics between LN+ and LN- patients are displayed in Table 4.

**Table 4:** Characteristics of patients with LN+ and LN- cervical cancer – breakdown of patient groups by histology and tumor stage

Characteristic		Lymph node positive patients	Lymph node negative patients
Histology - no. (%)	Squamous cell carcinoma	36 (90)	28 (82)
	Adenocarcinoma	3 (8)	4 (12)
	Adenosquamous	1 (3)	2 (6)
FIGO 2008 Stage - no. (%)	I	6 (15)	14 (41)
	II	19 (48)	16 (47)
	III	10 (25)	2 (6)
	IV	5 (13)	2 (6)

The primary tumor biopsies were collected at 3 different institutions: Stanford University (*n* = 47), Cancer Hospital of Shantou University Medical College (*n* = 10), and Santa Clara Valley Medical Center (*n* = 17). The tissue was collected from primary cervix tumor prior to the initiation of any therapy and was immediately put into RNAlater (Qiagen, Redwood City, California, USA). The cervical tumor samples were flash frozen in liquid nitrogen and stored at –80°C.

### Preparation of cDNA libraries for next-generation sequencing

For each tumor sample, total cDNA libraries for next-generation sequencing were prepared from RNA samples extracted from the tumor samples using the Qiagen AllPrep DNA/RNA Kit (Qiagen, Redwood City, California, USA). Each library was prepared using 1 μg of total RNA. This material was used to prepare sequencing libraries using the TruSeq™ RNA Sample Preparation kit (v2) from Illumina Proprietary (Illumina Inc., San Diego, CA, USA). Verification of cDNA library quality was performed using the high sensitivity DNA assay run provided by the protein and nucleic acid facility at Stanford University (Stanford University, Stanford, CA). The quality of the genomic product contained in the libraries was assessed with an Agilent 2100 Bioanalyzer (Agilent Technologies, Santa Clara, California, USA) that determined the sizing and quantification of the DNA fragments and smears. The libraries were sequenced using an Illumina HiSeq sequencer using the manufacture’s standard protocols at the Stanford Functional Genomics Facility (Stanford University, Stanford, CA). The first 24 samples were sequenced using the NextSeq GA single end read at 75 base pairs per cycle. The subsequent 50 samples were sequenced using the NextSeq GA paired end read at 150 base pairs per cycle.

### Alignment of mRNA next-generation sequencing reads

RNA sequencing reads were processed from raw FASTQ files and analyzed using STAR 2.3.0. Reads were filtered using the quality control program FASTQC (http://www.bioinformatics.babraham.ac.uk/projects/fastqc/) and subsequently trimmed using the FASTX-Toolkit (http://hannonlab.cshl.edu/fastx_toolkit/). The quality control plots were checked, and a file with unique reads and their corresponding counts was generated for each sample. The final processed unique reads were mapped with STAR to the human reference genome (https://code.google.com/p/rna-star/). Mapped reads were aligned and counted based on genomic annotations using Samtools (http://samtools.sourceforge.net/), and the reads were converted to counts using HTSeq (Version 0.9.1).

### Analysis

Genes without > 10 counts among > 7 samples (~10% of all samples) were filtered out from downstream analysis, eliminating extremely lowly expressed genes. Normalization was performed with DESeq2 using default parameters [19]. Variance Stabilizing Transformation was implemented to mitigate excessive dispersion in genes with low read counts for visualization and model development.

The patient samples from Stanford Medical Center and Cancer Center of Shantou Medical Center were used as a training cohort of 57 samples; the 17 samples from patients from Santa Clara Valley Medical Center were used as the test cohort. The training and test cohorts each have a nearly even distribution of LN+ and LN- samples. Standard scaling was performed on the training set, and the scaling factors derived from the training set were subsequently used to transform the test set.

Using the training set, differential expression between the LN+ and LN- samples was performed in DESeq2. The normal shrinkage estimator was used for effect size log fold-change shrinkage, and Benjamini-Hochberg False Discovery Rate (FDR) *p*-value adjustment was performed. Genes that displayed > 1.5 fold-change and ≤ 0.01 FDR adjusted *p*-value were considered differentially expressed. The stringent FDR adjusted *p*-value threshold was selected to minimize the likelihood of false positive identification of differentially expressed genes.

To determine which differentially expressed genes to include as candidates in the lymph node predictive model, the RandomForestClassifier package (Python Scikit-learn) was implemented with 1000 trees on the training set for forward selection using the greedy algorithm [20].

The final selection of candidate genes was performed in two steps. First, we excluded the genes with unknown biological function as previous data had suggested improved performance of a model by using previous knowledge about biology [21, 22]. Second, a Random Forest model was fit to all possible subsets and the model with the highest predictive accuracy for the training set was selected as the final model.

To build the models, Random Forest was used with 1000 trees and 10-fold cross-validation. For comparison, the genes included in the final Random Forest model were used to train additional classifiers based on Support Vector Machine with RBF kernel and Gaussian Naïve Bayes method. The final Random Forest model was evaluated using the separate test cohort, and the accuracy was calculated as the proportion of patients with lymph node status correctly classified.

Quantifications and analyses were performed using Python version 3.6.3 and R version 3.5.0. Statistical learning was performed with the Python package scikit-learn version 0.19.1. Plots and graphs were produced using the Python packages Matplotlib version 2.2.2 and Seaborn version 0.9.0.
